# Effects of drivers’ cell phone use on traffic safety: a systematic review

**Published:** 2019-08

**Authors:** Mirbahador Yazdani, Saber Azami-Aghdash, Mahsa Jafari, Mohammad Saadati, Vahideh Sadeghi

**Affiliations:** ^*a*^Road Traffic Injury Research Center, Tabriz, Iran.; ^*b*^Tabriz University of Medical Sciences, Tabriz, Iran.; ^*c*^Iran University of Science & Technology, Iran.

## Abstract

**Background:**

Cell phone use while driving or walking is a big challenge in traffic safety, worldwide. The aim of this study was to systematically identify the effect of cell phone usage on traffic crashes.

**Methods:**

A systematic search was performed in PubMed, Scopus, Google Scholar, Sid, IranMedix, Cochrane Library, Science Direct and Web of Knowledge. The included articles are published in 2001-2018. Besides searching in mentioned databases, hand searching was also done for identifying more related articles. After extracting related articles, they were assessed by using existing check lists.

**Results:**

Out of 1194 retrieved articles, 80 articles are included in this review. The included articles study different aspects of the cell phone usage effect on traffic safety, e.g. the effect of using cell phone on crash risk, crash severity, pedestrian crash, crash type, the effect of different cell phone use legislations on crash rates and fatalities in which different analytical methods (logistic regression, linear regression, structural equation modeling, econometric methods, etc.) have been used. The results of the studies in crash type show significant effects of cell phone usage on rear-end collisions, most of the studies in crash risk indicate higher collision risks for drivers who use cell phones and most of the studies on pedestrian crash show unsafe crossing behavior for cell phone users. Moreover, the results of hand held cell phone usage ban vary in the influence on traffic safety in different countries.

**Conclusions:**

Most of the included articles revealed that safety of drivers, cyclists and pedestrians on the road is undermined by cell phone usage. Therefore, efforts should be made on preventing distracted driving or walking which is one of the main contributors for road traffic injuries.

**Keywords:**

Cell phone, Driver, Pedestrian, Crash, Safety

